# Clear cell renal cell carcinoma molecular variations in non‐Hispanic White and Hispanic patients

**DOI:** 10.1002/cam4.5929

**Published:** 2023-04-20

**Authors:** Ken Batai, Yuliang Chen, Brenna A. Rheinheimer, Amit Arora, Ritu Pandey, Ronald L. Heimark, Erika R. Bracamonte, Nathan A. Ellis, Benjamin R. Lee

**Affiliations:** ^1^ Department of Cancer Prevention and Control Roswell Park Comprehensive Cancer Center Buffalo New York USA; ^2^ Department of Epidemiology and Biostatistics University of Arizona Tucson Arizona USA; ^3^ Department of Nutritional Sciences University of Arizona Tucson Arizona USA; ^4^ Department of Cellular and Molecular Medicine University of Arizona Tucson Arizona USA; ^5^ Department of Surgery University of Arizona Tucson Arizona USA; ^6^ Department of Pathology University of Arizona Tucson Arizona USA; ^7^ Department of Urology University of Arizona Tucson Arizona USA

**Keywords:** gene expression and diversity Hispanics and European Americans (comparison), health disparities, molecular subtype, *VHL*

## Abstract

**Background:**

The United States is becoming increasingly diverse, but few molecular studies have assessed the progression of clear cell renal cell carcinoma (ccRCC) in diverse patient populations. This study examined ccRCC molecular variations in non‐Hispanic White (NHW) and Hispanic patients and their effect on the association of gene expression with high‐grade (Grade 3 or 4) ccRCC and overall mortality.

**Methods:**

A total of 156 patients were included in *VHL* sequencing and/or TempO‐Seq analysis. DESeq2 was used to identify the genes associated with high‐grade ccRCC. Logistic regression analysis was performed to assess whether race and ethnicity was associated with high/moderate impact *VHL* somatic mutations and the ccA/ccB subtype. Cox regression analysis was performed to assess association of molecular subtype and gene expression with overall mortality.

**Results:**

NHWs had moderate or high impact mutations in the *VHL* gene at a higher frequency than Hispanics (40.2% vs. 27.4%), while Hispanics had a higher frequency of the ccA subtype than NHWs (61.9% vs. 45.8%). ccA was more common in patients with BMI≥35 (65.2%) than in those with BMI < 25 (45.0%). There were 11 differentially expressed genes between high‐ and low‐grade tumors. The *Haptoglobin* (*HP*) gene was most significantly overexpressed in high‐ compared to low‐grade ccRCC in all samples (*p*‐adj = 1.7 × 10^−12^). When stratified by subtype, the 11 genes were significantly differentially expressed in the ccB subtype, but none of them were significant after adjusting for multiple testing in ccA. Finally, patients with the ccB subtype had a significantly increased risk of overall mortality (HR 4.87; *p* = 0.01) compared to patients with ccA, and patients with high *HP* expression and ccB, had a significantly increased risk of mortality compared to those with low *HP* expression and ccA (HR 6.45, *p* = 0.04).

**Conclusion:**

This study reports ccRCC molecular variations in Hispanic patients who were previously underrepresented.

## Graphical abstract

Clear cell renal cell carcinoma is an obesity‐related subtype of kidney cancer which is unevenly affecting racial and ethnic minority groups. Hispanics have different molecular characteristics from non‐Hispanic Whites, potentially due to different prevalence of risk factors. Molecular variations likely impact use of molecular biomarkers to assess disease progression in diverse patient populations.

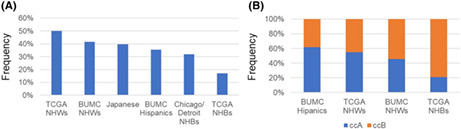

## 1 INTRODUCTION

The United States is becoming increasingly diverse, and the shift toward a precision approach for oncology care is accelerating, but the inclusion of patients from diverse backgrounds has been challenging.^1^, ^2^, ^3^ Hispanics have higher incidence and mortality rates of renal cell carcinoma (RCC) than non‐Hispanic Whites (NHWs).^4^, ^5^ Mexican Americans, particularly US‐born Mexican Americans, have elevated mortality rates even after accounting for neighborhood‐level socioeconomic status and treatment disparities.^6^, ^7^

Obesity rates are higher in Mexican Americans than in NHWs,^8^ and the obesity‐related histologic subtype, clear cell RCC (ccRCC), is more common in Hispanic RCC patients than in other racial and ethnic groups.^9^, ^10^ The papillary type, on the other hand, is more common in patients of African ancestry. There may also be great tumor molecular variations among RCC patients from different populations.^11^, ^12^ However, Hispanics and other racial and ethnic minority groups are underrepresented in RCC molecular studies, and the tumor characteristics and molecular variations among them are still unknown.^4^ Molecular biomarkers identified mainly in populations of European descent may not accurately predict disease progression or treatment response in patients from other racial and ethnic backgrounds.

This study explored ccRCC molecular variations in NHW and Hispanic patients and examined how molecular heterogeneity among patients affects the association of gene expression with high‐grade RCC and overall mortality. We focused on mutations in the *VHL* gene, which are most commonly altered in ccRCC, and in ccA/ccB,^13^ which have been shown to predict the risk of mortality.^14^, ^15^, ^16^ The ccA/ccB subtype can be assigned using a 34 gene panel, so it has potential clinical utility if validated in diverse patient populations. Our goals were: (1) to assess the differences in frequencies of *VHL* mutations and ccA/ccB subtype across racial and ethnic groups, and (2) to assess the ability of these biomarkers to predict mortality.

## 2 MATERIALS AND METHODS

### 2.1 Patient samples and data

Demographic, clinical, and pathological information of ccRCC patients who underwent nephrectomy for RCC treatment at Banner University Medical Cancer, Tucson, was collected from the electronic medical records. The characteristics of RCC patients have been previously described.^17^, ^18^ Race and ethnicity information reported in the medical records was used. Tumor and normal formalin‐fixed paraffin‐embedded (FFPE) surgical specimens were obtained. H&E‐stained slides, 2‐mm punches, and 10‐μm sections were obtained for each tumor and normal FFPE sample. This retrospective research protocol was approved by the institutional review board of the University of Arizona.

### 2.2 *VHL* (von Hippel–Lindau tumor suppressor) gene sequencing ccRCC tumor and normal samples

Three exons of *VHL* gene were screened for germline and somatic mutations in 151 patients. DNA was isolated from FFPE core punches using the ReliaPrep™ FFPE gDNA Miniprep System (Promega). DNA quantity was checked using a Qubit™ 2.0 instrument and a Qubit™ dsDNA HS Assay Kit (Thermo Fisher Scientific). All samples were found to have enough quantity to proceed to amplicon generation. Amplicons were designed based on Moore et al.^19^ (Table S1), and the touchdown PCR conditions for three amplicons were optimized. The amplicons were then generated for all samples for all three optimized amplicons. The amplicons were further dual‐indexed using Nextera XT Index Kit v2 Set A (Illumina). The indexed amplicons from each sample were quantified and pooled for equimolarity. The pool was assessed for quantity using the KAPA Library Quantification Kit on a Roche Light Cycler 480 platform. The pool was assessed for quality using Advanced Analytical Technology, Inc. Fragment analyzer with high‐sensitivity ngs fragment analysis kit. The pooled samples were diluted to a 2 nM concentration and sequenced on an Illumina MiSeq platform using a MiSeq Reagent Kit v2 (500 cycles). The results were demultiplexed and trimmed to create the FASTQ files for each sample.

### 2.3 TempO‐Seq analysis ccRCC tumor and normal samples

A total of 96 patients were included in the gene expression analysis using TempO‐Seq, transcriptome sequencing of approximately 20,000 protein‐coding genes (BioSpyder Technologies Inc.). The sample preparation methods have been described previously.^20^ Briefly, TempO‐Seq is an extraction‐free method. Samples (4–16 mm^2^ from the FFPE section) were added to the lysis reagent and detector oligos (Dos) for the genes to be measured, which were ligatable after annealing. Next, proteinase K was added. Hybridization exonuclease was then added to destroy the unhybridized Dos, followed by a single polymerase chain reaction. The samples were barcoded and pooled into a sequencing library. The sequencing library was purified using a Clontech NucleoSpin Gel and PCR Cleanup Kit (Clontech). Sequencing was performed using Illumina NextSeq. Demultiplexed FASTQ files were set as the sequencer outputs.

### 2.4 Bioinformatic analysis

*VHL* gene sequencing reads were aligned to the human genome (GRCh37) using BWA version 0.2.12,^21^ and base quality scores were recalibrated using the Genome Analysis Toolkit (GATK) version 3.7–0.^22^ Using sequence data from normal tissue samples, variant calling was performed to identify germline mutations using HaplotypeCaller in GATK version 4. Somatic variants were called for each tumor/normal pair using MuTect2^23^ in the same GATK package. Variants found in normal samples were included as a “panel of normal.” All identified variants were visually inspected using Integrative Genomics Viewer.^24^ Variants were annotated with SnpEff version 4.3 t^25^ using gene models from Ensembl release 75. A high‐confidence variant set was created from calls with at least 3% variant allele frequency in the tumor sample and no warning flags from MuTect2.

TempO‐SeqR was used to align demultiplexed FASTQ files, produce a read count for all samples in the sequencing run, and perform other steps of normalization, averaging of replicates, and calculating statistics for technical replicates. A modified centroid‐based approach with 34 genes was used to assign the molecular subtypes ccA and ccB.^14^, ^20^

### 2.5 Statistical analysis

The patients were characterized using means and frequencies. A two‐sample *t*‐test or analysis of variance (ANOVA) was used to test differences in means, and the chi‐squared test was used to test differences in frequencies of categorical variables. Logistic regression analysis was performed to assess whether race and ethnicity and behavioral characteristics were associated with the presence of high/moderate impact *VHL* somatic mutations and the ccA/ccB subtype. The DESeq2 differential expression algorithm^26^ was used to identify differentially expressed genes between high‐ and low‐grade ccRCC (Grade 3 and 4 vs. 1 and 2). The Benjamini and Hochberg procedure was used to adjust for multiple tests.^27^ Cox regression models were used to assess whether the molecular subtype, *VHL* mutations, and gene expression were associated with overall mortality after adjusting for age (<50 vs. ≥50), sex, race and ethnicity (Hispanic vs. NHW), diabetes, Hypertension, BMI (BIM ≥30 vs. <30), smoking history (have smoked vs. never smoked), grade (high vs. low), and stage (advanced vs. early). Associations between the expression of the identified genes and overall mortality were first examined using gene expression alone as a binary variable (high vs. low expression based on median gene expression). We then created categories combining high/low gene expression and the ccA/ccB subtype. The Human Pathology Atlas within The Human Protein Atlas was used to assess associations between identified genes and overall mortality in The Cancer Genome Atlas (TCGA) ccRCC (KIRC) dataset.^28^

## 3 RESULTS

### 3.1 Characteristics of ccRCC patients

A total of 156 patients with ccRCC were included in the *VHL* sequencing and/or TempO‐Seq analyses, 91 patients for both *VHL* sequencing and TempO‐Seq, 60 patients for *VHL* sequencing only, and 5 patients for TempO‐Seq only. Table 1 shows the characteristics of the patients included in the study. Hispanics were well represented in our study (42.3%) and showed characteristics different from those of NHWs. Compared to NHWs, Hispanics were younger (mean age, 55.7 vs. 61.2) and had a slightly higher mean Body Mass Index (BMI, 32.1 vs. 30.3). Hispanics were also more likely to have diabetes than NHWs were (43.9% vs. 26.2%; *p* = 0.03). More Hispanics reported that they never smoked than NHWs (69.7% vs. 47.6%; *p* = 0.01).

**TABLE 1:** Characteristics of ccRCC patients included for molecular study.

	NHW	Hispanic	Others/unknown	*p*
*n* (%)	84 (53.8)	66 (42.3)	6 (3.8)	
Age, mean (SD)	61.24(11.7)	56.1 (10.5)	49.8 (9.4)	0.002
BMI, mean (SD)	30.4 (6.9)	32.0 (8.7)	33.7 (7.6)	0.34
Sex, *n* (%)				0.25
Female	23 (27.4)	25 (37.9)	3 (50.0)	
Male	61 (72.6)	41 (62.1)	3 (50.0)	
Smoking history, *n* (%)				0.07
No	40 (47.6)	46 (69.7)	3 (50.0)	
Former	31 (36.9)	11 (16.7)	2 (33.3)	
Current	13 (15.5)	9 (13.6)	1 (16.7)	
Hypertension, *n* (%)				0.60
No	26 (31.0)	23 (34.8)	3 (50.0)	
Yes	58 (69.0)	43 (65.2)	3 (50.0)	
Diabetes, *n* (%)				0.06
No	62 (73.8)	37 (56.1)	3 (50.0)	
Yes	22 (26.2)	29 (43.9)	3 (50.0)	
Grade, *n* (%)				0.43
1 or 2	31 (36.9)	31 (47.0)	3 (50.0)	
3 or 4	53 (63.1)	35 (53.0)	3 (50.0)	
TNM Stage, *n* (%)				0.83
I or II	48 (57.1)	36 (54.5)	4 (66.7)	
III or IV	36 (42.9)	30 (45.5)	2 (33.3)	

### 3.2 *VHL* gene mutations variation

Three exons of the *VHL* genes were successfully sequenced in 151 patients to screen for germline mutations. Among the 151 patients, five NHW (5.9%) and one Hispanic (1.6%) patients had germline mutations in the coding region (Table S2). All were heterozygous for the identified variants. The mean age of these six patients was 62.2 (SD ±12.2) and was not significantly different from that of patients without germline mutations. Two NHW patients had an early diagnosis (age, 48 years). These patients were included in the subsequent analyses.

Of the 151 patients who were screened for somatic mutations, 73 somatic mutations in the coding regions of *VHL* were found in 56 patients (37.3%), and 11 patients had more than two somatic mutations in the coding regions (Table S3). Two patients had low‐ and high‐impact mutations in the introns (splice region and splice acceptor). These two patients were included in the following analyses because the splice acceptor variant had a high impact, while the other patient had high‐impact mutations in a coding region.

**FIGURE 1:**
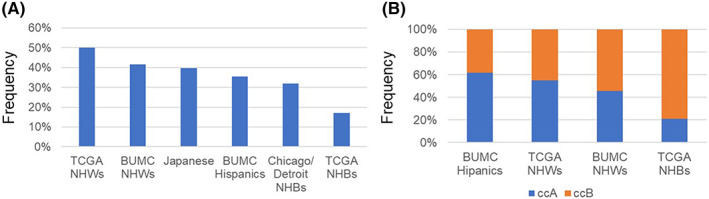
Frequency of *VHL* somatic mutations (A) and ccA/ccB molecular subtype (B) compared to TCGA NHWs and NHBs,^11^ Japanese^29^ and Chicago/Detroit NHBs.^12^

Mutations in the coding regions were more common in NHWs (41.5%) than in Hispanics (35.5%) (Figure 1A). However, the difference was not statistically significant (*p* = 0.49). When we focused on moderate‐ or high‐impact mutations, NHWs had mutations at a higher frequency than Hispanics (40.2% vs. 27.4%, Table S4). In the logistic regression model, Hispanics showed a trend toward reduced odds of having high/moderate impact mutations (Table 2; *p* = 0.07) after adjusting for age and sex. Although not statistically significant, the frequencies of moderate/high‐impact somatic mutations were also higher among former (35.7%) and current smokers (43.5%) than among non‐smokers (30.6%) and among normal weight (37.5%) and overweight (38.2%) patients than among obese patients (30.4%). After including smoking history and BMI in the regression model, Hispanic ethnicity was not associated with the presence of high/moderate‐impact somatic mutations (*p* = 0.13). Including grade and stage in the regression models did not change the results.

**TABLE 2:** Assessing association between Hispanic ethnicity and presence of moderate and high impact mutations.

	Model 1		Model 2	
	OR (95% CI)	*p*	OR (95% CI)	*p*
Age				
<50	Reference		Reference	
≥50	1.35 (0.61–2.99)	0.46	1.33 (0.59–3.01)	0.49
Sex				
Female	Reference		Reference	
Male	0.56 (0.27–1.17)	0.12	0.50 (0.23–1.08)	0.08
Race/ethnicity				
NHW	Reference		Reference	
Hispanic	0.50 (0.24–1.02)	0.07	0.58 (0.27–1.23)	0.15
Other/unknown	0.22 (0.02–2.09)	0.12	0.25 (0.03–2.37)	0.23
Smoking				
No			Reference	
Current/Former			1.52 (0.72–3.22)	0.27
BMI				
<30			Reference	
≥30			0.62 (0.30–1.29)	0.20

### 3.3 ccA versus ccB molecular subtype variation

The ccA/ccB molecular subtype was assessed using the TempO‐Seq data. A comparison of the frequencies of ccA/ccB molecular subtypes between Hispanics and NHWs revealed a higher frequency of the ccA subtype in Hispanics than in NHWs (61.9% vs. 45.8%; Figure 1B). The ccA subtype was also more common in patients with a BMI≥35 (63.6%) than in those with a BMI < 25 (47.8%) (Table S5). ccB was more common in patients with diabetes than in those without (67.6% vs. 33.9%; *p* = 0.002). In the model adjusted for age and sex, Hispanics had statistically non‐significant increased odds of having ccA (Table 3). After adjusting for additional factors (smoking history, diabetes, and BMI) in Model 2, Hispanics had significantly increased odds of having ccA (OR, 2.95; 95% CI: 1.06–8.23). Further including grade and stage in the regression models slightly attenuated the association for Hispanics (OR 2.80; 95% CI: 0.99–7.93) but did not change the overall results.

**TABLE 3:** Odds of having ccA subtype (*n* = 96).

	Model 1		Model 2	
	OR (95% CI)	*p*	OR (95% CI)	*p*
Age				
<50	Reference		Reference	
≥50	1.68 (0.66–4.31)	0.28	1.10 (0.37–3.25)	0.86
Sex				
Female	Reference		Reference	
Male	0.86 (0.35–2.12)	0.74	0.77 (0.28–2.16)	0.62
Race/ethnicity				
NHWs	Reference		Reference	
Hispanics	1.89 (0.81–4.42)	0.14	2.95 (1.06–8.23)	0.04
Other/unknown	1.86 (0.29–11.76)	0.51	3.45 (0.42–28.37)	0.25
Smoking history				
No			Reference	
Former/current			1.31 (0.49–3.52)	0.59
Diabetes				
No			Reference	
Yes			0.15 (0.05–0.45)	<0.001
BMI				
<25			Reference	
≥25, <30			1.52 (0.44–5.27)	0.51
≥30, <35			1.63 (0.43–6.18)	0.48
≥35			2.54 (0.63–10.19)	0.19

### 3.4 Genes differentially expressed between high‐ and low‐grade ccRCC

To evaluate whether tumor heterogeneity potentially affects the prediction of prognosis, we first performed differential expression analysis. There were 19 differentially expressed genes with *p*_adj_ <0.05 (Figure S1), and 12 of them had log_2_ fold change <−1.0 or >1.0 (Table S6) in the analysis with all the patients combined. All of them, except for *UCHL1*, were overexpressed in high‐grade tumors compared to low‐grade tumors and in tumors compared to adjacent normal tissues.

Then, separate analyses were performed for ccA and ccB subtypes to assess whether these 11 genes (not including *UCHL1* that had a negative log_2_ fold change in tumors compared to normal tissues) were differentially expressed between high‐ and low‐grade tumors, similarly in ccA and ccB. These 11 genes were significantly differentially expressed among patients with ccB subtype. In patients with ccA subtype, none of these genes were significant after adjusting for multiple testing. For example, the *Haptoglobin* (*HP*) gene was most significantly overexpressed in high‐grade compared to low‐grade ccRCC in an analysis including all samples (log_2_ fold change 4.0, *p*_adj_ = 1.7×10^−12^). *HP* was significantly overexpressed in high‐grade compared to low‐grade ccRCC in ccB (log_2_ fold change 2.5, *p*_adj_ = 0.03), but not in ccA (log_2_ fold change 1.9, *p*_adj_ = 0.17). We also examined the heterogeneity in the association between Hispanics and NHWs. In Hispanics, only *FGG* was significantly overexpressed, while *HP*, *SAA2*, and *BIRC3* were significant in NHWs with *p*_adj_ <0.05 (Table S7).

### 3.5 Heterogeneities in associations of gene expression with overall mortality

**FIGURE 2:**
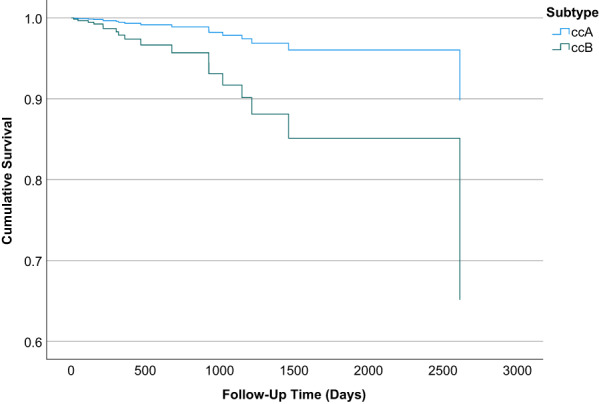
Patients with ccB subtype have elevated overall mortality risk compared to patients with ccA.

Finally, we assessed whether the ccA/ccB subtype and the identified genes were associated with an increased risk of mortality. Patients with ccB subtype had a significantly increased risk of overall mortality (HR 4.87; 95% CI: 1.37–17.31) compared to patients with ccA subtype (Figure 2; Table S8). Having moderate/high‐impact *VHL* somatic mutations were significantly associated with overall mortality when ccA/ccB subtype was included in the model (Table S9). In these models, we did not observe statistical differences in risk of mortality between NHW and Hispanics. Obese patients and patients with hypertension had a reduced risk of mortality. When we examined the combined effect of subtype and race and ethnicity, compared to Hispanics with ccA subtype, Hispanic with ccB had statistically significant increased risk of mortality (HR 4.47; 95% CI: 1.01–19.69). The effect of subtype was slightly stronger for NHWs with HR 6.29 (95% CI: 1.17–33.76) than for Hispanics with ccA subtype.

Among the identified genes associated with high‐grade ccRCC, higher mRNA expression of all genes, except *FGG*, was significantly associated with an increased risk of overall mortality in the TCGA KIRC dataset (Figure S2). In our dataset, high expression of *DHCR24* was associated with an increased risk of overall mortality. No other genes were associated with overall mortality (Table S10).

We then examined the combined effect of subtype and gene expression using the low expression of each gene in ccA as a reference. Only *DHCR24* yielded the expected results. Compared to ccA/*DHCR24*‐low, ccA/*DHCR24*‐high, ccB/*DHCR24*‐low, and ccB/*DHCR24*‐high had HR of 2.29, 3.81, and 8.05, respectively. Only *HP* showed significantly elevated risk of mortality in both high and low expression in ccB (HR 10.94 and 6.45 for ccB/*HP*‐low and ccB/*HP*‐high) compared to ccA/*HP*‐low. Low expression of *SAA2*, *QSOX1*, and *LOX* in ccB also showed a significantly increased risk of mortality compared to low expression of these genes in ccA. Taken together, our analysis showed that the identified genes were heterogeneously associated with overall mortality between the ccA and ccB subtypes.

## 4 DISCUSSION

The Unites States is becoming increasingly diverse, but few molecular studies have assessed RCC progression in diverse patient populations. This study showed that Hispanic and NHW tumors have different molecular characteristics, potentially owing to the differences in the prevalence of behavioral risk factors. The frequencies of molecular subtypes were different in Hispanics and NHWs, and the effects of genes associated with aggressive RCC and mortality differed between the subtypes. Different molecular characteristics in racial and ethnic groups may affect the clinical utility of the identified biomarkers and clinical treatments in diverse patient populations.

Our study findings add to the literature by showing potential ccRCC molecular variations across diverse populations. In general, *VHL* somatic mutations are more common in NHWs.^11^ They are less common in Hispanics and Japanese, but more common than in non‐Hispanic Blacks (NHBs).^12^, ^29^ The ccA subtype is most common in Hispanics and least common in NHBs.^11^ Molecular differences across racial and ethnic groups have also been reported for other cancer types.^30^, ^31^, ^32^ This illustrates the challenges in the use of molecular biomarkers to assess disease progression in racially ethnically diverse patient populations as well as the necessity of including patients from racially ethnically diverse backgrounds in RCC molecular studies.

Moreover, the associations of *VHL* somatic mutations and ccA/ccB subtypes with race and ethnicity changed after adjusting for behavioral factors, such as obesity and smoking, suggesting that these factors potentially affect molecular characteristics. Smoking is known to cause somatic mutations and a high tumor mutational burden in many cancer types including RCC.^33^, ^34^ While no link between smoking and *VHL* mutations has been reported, one study reported that associations between high vegetable and citrus fruit intake and reduced odds of *VHL* mutations among smokers.^35^ Previous studies have shown that the high BMI is associated with ccA,^15^, ^16^ and Hispanics, particularly Mexican Americans, have a higher BMI than NHWs.^36^ The prevalence of risk factors, such as smoking and obesity, may vary across racial and ethnic groups and may be the underlying factors influencing RCC molecular variation.

We expected to find a more aggressive subtype in Hispanics in Arizona who have higher risk of mortality.^6^ In contrast, ccB, which is less common in Hispanics, was associated with an increased risk of overall mortality in this previous studies.^14^, ^15^, ^16^ ccA/ccB subtypes developed mainly based on NHWs may not be applicable to Hispanics for assessing RCC progression. Alternatively, the Hispanics included in this study were not representative of Hispanics in Arizona, and we did not find a statistically significant difference in the risk of mortality between Hispanics and NHWs. Nonetheless, the ccB subtype drove gene expression signatures in high‐grade ccRCC in our dataset supporting ccB as a more aggressive subtype. Among the identified genes, *HP*, *DHCR24*, *SAA2*, *QSOX1*, and *LOX* were associated with an increased risk of mortality in patients with ccB. *HP* encodes the precursor of the alpha and beta chains of haptoglobin. The *HP* gene and haptoglobin are biomarkers of kidney disease risk^37^ metabolic diseases,^38^, ^39^ and other health conditions, including several cancer types.^40^, ^41^ *DHCR24* (24‐dehydrocholesterol reductase) is involved in cholesterol biosynthesis. The role of *DHCR24* is still not well understood, but genes involved in cholesterol metabolism and biosynthesis are dysregulated in ccRCC, and exogenous cholesterol promotes ccRCC growth.^42^ *DHCR24* has been implicated in several cancer types.^43^, ^44^, ^45^

Biomarkers to assess disease progression are often developed mainly based on the tumor samples from European descent patients. These biomarkers may not be clinical useful for patients from other backgrounds, and patients from diverse backgrounds need to be included to assess the utility and acceptability of these biomarkers.^1^, ^46^ This study assessed molecular variations as we plan for future RCC molecular biomarker studies in racially and ethnically diverse patient populations. Common molecular biomarkers, such as ccA/ccB subtype and *VHL* mutations, are potentially useful for predicting disease progression or therapeutic response in diverse patient populations. However, the prevalence and effects of these molecular biomarkers may vary across populations, and there may be more informative biomarkers for racial and ethnic minority groups who have elevated risk of RCC mortality.

This study has some potential limitations. The patients included in this study were treated at an academic hospital and may not have been representative of the local population. The hospital is in southern Arizona, and Hispanic Americans are mainly of Mexican descent in the Southwestern border region of the United States. Our findings may not be generalizable and studies in other parts of the United States, where other Hispanic subgroups, such as Cuban and Puerto Rican are better represented, may show different patterns. We previously reported different RCC clinicopathologic, surgical treatment, and outcome patterns across Hispanic subgroups.^6^, ^9^, ^47^ This study also explored the associations between modifiable risk factors and molecular data, but the associations were not significant, potentially due to the small sample size, small effects, or imprecise measurement/misclassification. Due to the small sample size, stratified analysis for Hispanics and NHWs using Cox regression analysis to assess the associations between identified genes and overall mortality was not possible. Finally, due to retrospective nature of this study, we were not able to collect potentially important information, such as detailed information on patients' comorbidities and health conditions, socioeconomic factors, and environmental exposures, which may have confounded the relationship between molecular subtype and mortality.^6^, ^16^, ^48^ Additional studies with a much larger sample size are needed (1) to further explore ccRCC molecular variations across different populations, (2) to identify unique molecular subtypes if there are, and (3) to assess the clinical utility and significance of molecular biomarkers predicting disease progression in diverse patient populations.

In conclusion, this study reported variations in ccRCC molecular characteristics by including Hispanic patients, who were previously underrepresented. However, the current study is exploratory. Future studies should include more patients from diverse backgrounds to fully understand ccRCC molecular variation across different populations, potential factors influencing the variation, and implications in biomarker studies of ccRCC.

## AUTHOR CONTRIBUTIONS

**Ken Batai:** Conceptualization (lead); data curation (equal); formal analysis (equal); funding acquisition (lead); writing – original draft (lead). **Yuliang Chen:** Data curation (equal); formal analysis (lead); writing – review and editing (equal). **Brenna A Rheinheimer:** Formal analysis (equal); writing – review and editing (equal). **Amit Arora:** Data curation (equal); formal analysis (equal); writing – review and editing (equal). **Ritu Pandey:** Investigation (equal); methodology (equal); writing – review and editing (equal). **Ronald L Heimark:** Investigation (equal); methodology (equal); writing – review and editing (equal). **Erika R Bracamonte:** Investigation (equal); writing – review and editing (equal). **Nathan A Ellis:** Conceptualization (supporting); methodology (equal); writing – review and editing (equal). **Benjamin R Lee:** Conceptualization (supporting); data curation (equal); funding acquisition (supporting); writing – review and editing (equal).

## FUNDING INFORMATION

This project was supported by the Department of Defense (W81XWH2110811), National Cancer Institute (P30CA023074), Urology Care Foundation Research Scholar Award, and Institutional Research Grant Number IRG‐16‐124‐37‐IRG from the American Cancer Society.

## CONFLICT OF INTEREST STATEMENT

The authors do not have conflict of interest to disclose.

## ETHICS STATEMENT

The retrospective research protocol was approved by the institutional review board of the University of Arizona.

## Supporting information


Data S1:

## Data Availability

Raw data for this study were generated at the University of Arizona Genetics Core and BioSpyder Technologies Inc.). Derived data and clinical data supporting the findings of this study are available from the corresponding author upon request.
