# Supplementary material for: Clear cell renal cell carcinoma molecular variations in non‐Hispanic White and Hispanic patients
Source: Cancer Med. 2023 Apr 20;12(11):12792–801. doi: 10.1002/cam4.5929 (PMC10278477; doi:10.1002/cam4.5929)

*Supplementary Material*

# Clear Cell Renal Cell Carcinoma Molecular Variations in Non-Hispanic White and Hispanic Patients

**Ken Batai*, Yuliang Chen, Brenna A. Rheinheimer, Amit Arora, Ritu Pandey, Ronald L. Heimark, Erika R. Bracamonte, Nathan A. Ellis, Benjamin R. Lee**

*** Correspondence:** Ken Batai, PhD: Ken.Batai@RoswellPark.org

**Supplementary Table 1** Primer sequences used to generate the amplicons

| Primer | Sequence |
|---|---|
| Exon 1- Forward | 5′-CTACGGAGGTCGACTCGG |
| Exon 1- Reverse | 5′-GCTTCAGACCGTGCTATCG |
| Exon 2- Forward | 5′-CCGTGCCCAGCCACCGGTGTG |
| Exon 2- Reverse | 5′-GGATAACGTGCCTGACATCAG |
| Exon 3- Forward | 5′-CGTTCCTTGTACTGAGACCCTAG |
| Exon 3- Reverse | 5′-GAACCAGTCCTGTATCTAGATCAAG |

**Supplementary Table 2** *VHL* germline mutations detected in 6 ccRCC patients

| Position | SNP ID | Ref | Alt | Effects | Mutations | AA Change |
|---|---|---|---|---|---|---|
| 10183534 | rs578091032 | G | C | start lost | c.3G>C | |
| 10183554 | rs1559425498 | G | T | missense | c.23G>T | p.Ala5Ser |
| 10183605 | rs35460768 | C | T | missense | c.74C>T | p.Pro25Leu |
| 10183732 | rs769658318 | C | G | missense | c.201C>G | p.Asn67Lys |
| 10191489 | rs730882035 | G | T | protein-protein contact & missense | c.482G>T | p.Arg161Leu |
| 10191541 | | G | C | synonymous | c.534G>C | |

Single Nucleotide Polymorphism (SNP), reference allele (Ref), alternative variant (Alt), Amio Acid Change (AA) Change

**Supplementary Table 3** *VHL* somatic mutations detected

| **Effect** | **n** | **Impact** |
|---|---|---|
| Synonymous | 13 | Low |
| Missense | 13 | Moderate |
| Frameshift | 24 | High |
| Protein-protein contact | 3 | High |
| Stop gained | 6 | High |
| Structural interaction | 14 | High |
| Splice acceptor (intron) | 1 | High |
| Splice region (intron) | 1 | Low |

**Supplementary Table 4** Moderate and high impact mutations and correlation with demographic and clinical characteristics

| | No mutation | Mutation | *p* |
|---|---|---|---|
| **Age, n (%)** | | | 0.70 |
| **<50** | 25 (62.5) | 15 (37.5)) | |
| **≥50** | 74 (67.3) | 36 (32.7) | |
| **Race/ethnicity, n (%)** | | | 0.18 |
| **NHW** | 49 (59.0) | 33 (40.2) | |
| **Hispanic** | 45 (72.6) | 17 (27.4) | |
| **Others/Unknown** | 5 (83.3) | 1 (16.7) | |
| **Sex, n (%)** | | | 0.20 |
| **Male** | 71 (69.6) | 31 (30.4) | |
| **Female** | 28 (58.3) | 20 (41.7) | |
| **Grade, n (%)** | | | 0.73 |
| **1 or 1** | 42 (67.7) | 20 (32.3) | |
| **3 or 4** | 57 (64.8) | 31 (35.2) | |
| **TNM Stage, n (%)** | | | 0.22 |
| **Early (I/II)** | 53 (61.6) | 33 (38.4) | |
| **Advanced (III/IV)** | 46 (71.9) | 18 (28.1) | |
| **Vital Status, n (%)** | | | 0.48 |
| **Alive** | 82 (65.1) | 44 (34.9) | |
| **Deceased** | 17 (73.9) | 6 (26.1) | |
| **Smoking History, n (%)** | | | 0.49 |
| **No** | 59 (69.4) | 26 (30.6) | |
| **Former** | 27 (64.3) | 15 (35.7) | |
| **Current** | 13 (56.5) | 10 (43.5) | |
| **Hypertension, n (%)** | | | 0.37 |
| **No** | 31 (60.8) | 20 (39.2) | |
| **Yes** | 68 (68.7) | 31 (31.3) | |
| **Diabetes, n (%)** | | | 0.86 |
| **No** | 65 (65.0) | 35 (35.0) | |
| **Yes** | 34 (68.0) | 16 (32.0) | |
| **BMI, n (%)** | | | 0.69 |
| **<25** | 17 (60.7) | 11 (39.3) | |
| **≥25, <30** | 31 (62.0) | 19 (38.0) | |
| **≥30, <35** | 28 (71.8) | 11 (28.2) | |
| **≥35** | 23 (69.7) | 10 (31.3) | |

**Supplementary Table 5** ccA/ccB molecular subtype and correlation with demographic and clinical characteristics

| | ccA | ccB | *p* |
|---|---|---|---|
| **Age** | | | 0.26 |
| **<50** | 18 (64.3) | 10 (35.7) | |
| **≥50** | 34 (50.0) | 34 (50.0) | |
| **Race/ethnicity** | | | 0.26 |
| **NHW** | 22 (45.8) | 26 (54.2) | |
| **Hispanic** | 26 (61.9) | 16 (38.1) | |
| **Others** | 4 (66.7) | 2 (33.3) | |
| **Sex** | | | 0.66 |
| **Male** | 35 (52.2) | 32 (47.8) | |
| **Female** | 17 (58.6) | 12 (41.4) | |
| **Grade** | | | 0.84 |
| **1 or 1** | 23 (56.1) | 18 (43.9) | |
| **3 or 4** | 29 (52.7) | 26 (47.3) | |
| **TNM Stage** | | | 0.99 |
| **Early (I/II)** | 27 (54.0) | 23 (46.0) | |
| **Advanced (III/IV)** | 25 (54.3) | 21 (45.7) | |
| **Vital Status** | | | 0.12 |
| **Alive** | 45 (57.7) | 33 (42.3) | |
| **Dead** | 7 (38.9) | 11 (61.1) | |
| **Smoking History** | | | 0.95 |
| **No** | 30 (53.6) | 26 (46.4) | |
| **Former** | 15 (53.6) | 13 (46.4) | |
| **Current** | 7 (58.3) | 5 (41.7) | |
| **Hypertension** | | | 0.83 |
| **No** | 15 (51.7) | 14 (48.3) | |
| **Yes** | 37 (55.2) | 30 (44.8) | |
| **Diabetes** | | | **0.002** |
| **No** | 41 (66.1) | 21 (33.9) | |
| **Yes** | 11 (32.4) | 23 (67.6) | |
| **BMI** | | | 0.72 |
| **<25** | 11 (47.8) | 12 (52.2) | |
| **≥25, <30** | 15 (55.6) | 12 (44.4) | |
| **≥30, <35** | 12 (50.0) | 12 (50.0) | |
| **≥35** | 14 (63.6) | 8 (36.4) | |
| ***VHL* Somatic Mutations** | | | 0.41 |
| **High impact** | 7 (43.8) | 9 (56.3) | |
| **Other mutations or no mutation** | 39 (57.4) | 29 (42.6) | |

**Supplementary Table 6** 11 genes from differential expression analysis comparing high vs. low grade ccRCC with $log_2$ fold change <-1.0 or >1.0 and p-adjusted <0.05

| | **All** | | | **ccA** | | | **ccB** | | |
|---|---|---|---|---|---|---|---|---|---|
| | **log2FC** | ***p*** | ***p-adj*** | **log2FC** | ***p*** | ***p-adj*** | **log2FC** | ***p*** | ***p-adj*** |
| ***HP*** | 3.994 | 1.17E-15 | 1.76E-12 | 1.952 | 0.0002 | 0.17 | 2.523 | 0.0006 | 0.03 |
| ***SAA2*** | 3.702 | 1.26E-08 | 9.44E-06 | 2.819 | 0.003 | 0.52 | 5.046 | 4.11E-09 | 4.40E-06 |
| ***FGG*** | 3.568 | 4.08E-08 | 2.04E-05 | 0.654 | 0.35 | 1.00 | 3.504 | 8.40E-05 | 0.01 |
| ***BIRC3*** | 1.454 | 5.64E-08 | 2.12E-05 | 0.020 | 0.95 | 1.00 | 2.899 | 2.54E-14 | 5.43E-11 |
| ***QSOX1*** | 1.541 | 3.10E-07 | 9.33E-05 | 0.467 | 0.13 | 1.00 | 1.994 | 1.38E-05 | 0.004 |
| ***C1S*** | 1.191 | 1.40E-06 | 0.0004 | 0.838 | 0.008 | 0.64 | 1.448 | 0.0001 | 0.013 |
| ***FGB*** | 2.815 | 1.16E-05 | 0.002 | 1.492 | 0.12 | 1.00 | 3.655 | 1.46E-05 | 0.004 |
| ***CXCL1*** | 1.507 | 0.0001 | 0.018 | -0.077 | 0.86 | 1.00 | 2.302 | 9.72E-05 | 0.01 |
| ***NRN1*** | 1.378 | 0.0002 | 0.02 | 0.373 | 0.39 | 1.00 | 1.757 | 0.0005 | 0.03 |
| ***DHCR24*** | 1.102 | 0.0002 | 0.02 | 0.215 | 0.53 | 1.00 | 1.067 | 0.002 | 0.08 |
| ***LOX*** | 1.174 | 0.0004 | 0.04 | 0.643 | 0.08 | 1.00 | 1.479 | 0.0065 | 0.16 |

**Supplementary Table 7** 11 genes from differential expression analysis comparing high vs. low grade ccRCC with $log_2$ fold change <-1.0 or >1.0 and p-adjusted <0.05 in Hispanics and NHWs

| | **Hispanics** | | | **NHWs** | | |
|---|---|---|---|---|---|---|
| | **log2FC** | ***p*** | ***p-adj*** | **log2FC** | ***p*** | ***p-adj*** |
| ***HP*** | 2.504 | 3.85E-05 | 0.06 | 5.195 | 2.91E-13 | 4.87E-09 |
| ***SAA2*** | 4.016 | 1.59E-04 | 0.18 | 4.025 | 4.39E-06 | 0.02 |
| ***FGG*** | 4.273 | 6.96E-08 | 0.0005 | 3.227 | 0.002 | 0.95 |
| ***BIRC3*** | 0.932 | 0.01 | 0.99 | 1.889 | 3.96E-06 | 0.02 |
| ***QSOX1*** | 1.392 | 5.02E-05 | 0.08 | 1.470 | 0.003 | 0.99 |
| ***C1S*** | 1.235 | 0.0008 | 0.42 | 1.375 | 0.0002 | 0.30 |
| ***FGB*** | 2.874 | 0.00098 | 0.46 | 2.599 | 0.01 | 0.99 |
| ***CXCL1*** | 0.729 | 0.22 | 0.99 | 1.420 | 0.006 | 0.99 |
| ***NRN1*** | 0.920 | 0.03 | 0.99 | 1.568 | 0.01 | 0.99 |
| ***DHCR24*** | 0.745 | 0.09 | 0.99 | 0.810 | 0.03 | 0.99 |
| ***LOX*** | 1.455 | 0.005 | 0.86 | 0.921 | 0.03 | 0.99 |

**Supplementary Table 8** Assessment of ccA/ccB subtype and race/ethnicity on overall mortality

| | HR 95% CI | *p* | HR 95% CI | *p* |
|---|---|---|---|---|
| **Race/ethnicity** | | | | |
| **NHW** | Reference | | | |
| **Hispanic** | 0.81 (0.25-2.61) | 0.72 | | |
| **Subtype** | | | | |
| **ccA** | Reference | | | |
| **ccB** | 4.87 (1.37-17.31) | 0.01 | | |
| **Race/ethnicity and subtype** | | | | |
| **Hispanic ccA** | | | Reference | |
| **Hispanic ccB** | | | 4.47 (1.01-19.69) | 0.048 |
| **NHW ccA** | | | 1.02 (0.13-7.99) | 0.98 |
| **NHW ccB** | | | 6.07 (1.16-31.93) | 0.03 |
| **Age** | | | | |
| **<50** | Reference | | Reference | |
| **≥50** | 4.28 (0.42-43.38) | 0.22 | 4.39 (0.43-44.82) | 0.21 |
| **Sex** | | | | |
| **Female** | Reference | | Reference | |
| **Male** | 2.10 (0.46-9.63) | 0.34 | 2.22 (0.45-10.93) | 0.33 |
| **BMI** | | | | |
| **<30** | Reference | | Reference | |
| **≥30** | 0.13 (0.02-0.92) | 0.04 | 0.13 (0.02-0.92) | 0.04 |
| **Hypertension** | | | | |
| **No** | Reference | | Reference | |
| **Yes** | 0.14 (0.03-0.55) | 0.005 | 0.14 (0.03-0.59) | 0.007 |
| **Diabetes** | | | | |
| **No** | Reference | | Reference | |
| **Yes** | 2.47 (0.68-8.96) | 0.17 | 2.55 (0.68-9.59) | 0.17 |
| **Ever Smoked** | | | | |
| **No** | | | Reference | |
| **Yes** | 0.39 (0.08-1.87) | 0.24 | 0.37 (0.07-1.87) | 0.23 |
| **Grade** | | | | |
| **Low** | Reference | | Reference | |
| **High** | 6.72 (0.74-61.38) | 0.09 | 6.82 (0.74-62.72) | 0.09 |
| **Stage** | | | | |
| **Early** | Reference | | Reference | |
| **Advanced** | 13.57 (1.25-147.16) | 0.03 | 14.30 (1.25-163.06) | 0.03 |

**Supplementary Table 9** Associations of *VHL* somatic mutations and ccA/ccB subtype with overall mortality

| | Model 1 | | Model 2 | | Model 3 | |
|---|---|---|---|---|---|---|
| | **HR (95% CI)** | ***p*** | **HR (95% CI)** | ***p*** | **HR (95% CI)** | ***p*** |
| **Moderate/High impact *VHL* mutations vs. no coding mutations** | 1.06 (0.38-2.96) | 0.92 | | | 6.93 (1.02-47.19) | **0.048** |
| **ccB vs. ccA subtype** | | | 4.87 (1.37-17.31) | **0.01** | 7.89 (1.10-56.63) | **0.04** |

Associations of *VHL* somatic mutations and subtype with overall mortality were respectively tested in Model 1 and Model 2 adjusting for age (<50 vs. ≥50), sex, race/ethnicity (Hispanic vs. NHW), diabetes, Hypertension, BMI (BIM ≥30 vs <30), smoking history (have smoked vs. never smoked), grade (high vs. low), and stage (advanced vs. early). Both *VHL* somatic mutations and subtype were included in Model 3.

**Supplementary Table 10** Assessment of heterogenous effect of identified genes on overall mortality in all the samples and combined with ccA/ccB subtype

| | All HR (95% CI) | *p* | ccA HR (95% CI) | *p* | ccB HR (95% CI) | *p* |
|---|---|---|---|---|---|---|
| ***HP*** | | | | | | |
| **Low** | Reference | | Reference | | 10.94 (1.55-77.44) | **0.02** |
| **High** | 2.20 (0.66-7.33) | 0.20 | 4.10 (0.56-29.98) | 0.16 | 6.45 (1.06-39.19) | **0.04** |
| ***SAA2*** | | | | | | |
| **Low** | Reference | | Reference | | 8.24 (1.19-57.00) | **0.03** |
| **High** | 1.05 (0.30-3.74) | 0.94 | 1.60 (0.17-15.05) | 0.68 | 4.41 (0.76-25.72) | 0.10 |
| ***FGG*** | | | | | | |
| **Low** | Reference | | Reference | | 1.27 (0.18-8.89) | 0.81 |
| **High** | 0.58 (0.17-2.02) | 0.39 | 0.16 (0.02-1.18) | 0.07 | 2.93 (0.56-15.33) | 0.20 |
| ***BIRC3*** | | | | | | |
| **Low** | Reference | | Reference | | 2.71 (0.44-16.61) | 0.28 |
| **High** | 0.51 (0.12-2.25) | 0.37 | 0.13 (0.02-1.09) | 0.06 | 1.83 (0.35-9.48) | 0.47 |
| ***QSOX1*** | | | | | | |
| **Low** | Reference | | Reference | | 12.58 (1.62-97.37) | **0.02** |
| **High** | 1.55 (0.43-5.52) | 0.50 | 0.90 (0.09-9.47) | 0.93 | 3.86 (0.85-17.63) | 0.08 |
| ***C1S*** | | | | | | |
| **Low** | Reference | | Reference | | 4.78 (0.72-31.8) | 0.11 |
| **High** | 0.71 (0.19-2.63) | 0.61 | 0.40 (0.05-2.95) | 0.40 | 2.63 (0.51-13.43) | 0.25 |
| ***FGB*** | | | | | | |
| **Low** | Reference | | Reference | | 2.82 (0.50-15.98) | 0.24 |
| **High** | 0.23 (0.06-0.91) | 0.04 | 0.23 (0.03-1.63) | 0.14 | 1.26 (0.17-9.63) | 0.83 |
| ***CXCL1*** | | | | | | |
| **Low** | Reference | | Reference | | 3.25 (0.55-19.35) | 0.20 |
| **High** | 0.74 (0.23-2.40) | 0.62 | 0.45 (0.07-2.89) | 0.40 | 3.18 (0.61-16.64) | 0.17 |
| ***NRN1*** | | | | | | |
| **Low** | Reference | | Reference | | 5.47 (0.77-38.95) | 0.09 |
| **High** | 1.33 (0.38-4.75) | 0.66 | 0.77 (0.11-5.18) | 0.78 | 3.92 (0.73-21.16) | 0.11 |
| ***DHCR24*** | | | | | | |
| **Low** | Reference | | Reference | | 3.81 (0.60-24.15) | 0.16 |
| **High** | 3.72 (1.04-13.36) | **0.04** | 2.29 (0.31-16.88) | 0.42 | 8.05 (1.63-39.67) | **0.01** |
| ***LOX*** | | | | | | |
| **Low** | Reference | | Reference | | 7.44 (1.08-51.42) | **0.04** |
| **High** | 1.12 (0.23-5.45) | 0.89 | 1.37 (0.13-14.86) | 0.80 | 4.89 (0.61-39.09) | 0.14 |

Adjusting for age (<50 vs. ≥50), sex, race/ethnicity (Hispanic vs. NHW), diabetes, Hypertension, BMI (BIM ≥30 vs <30), smoking history (have smoked vs. never smoked), grade (high vs. low), and stage (advanced vs. early).

**Supplementary Figure 1** Differential expression analysis results comparing high vs. low grade ccRCC.

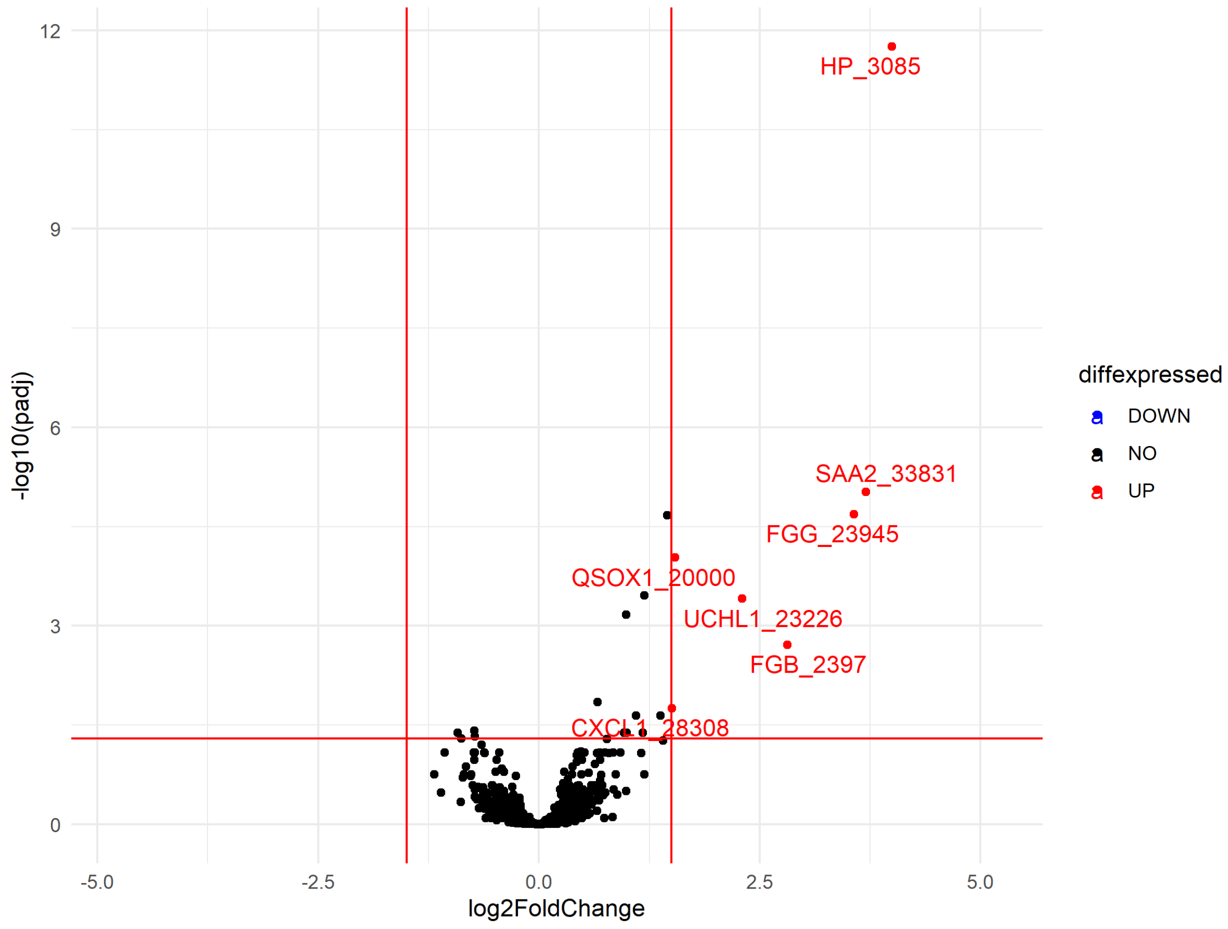

**Supplementary Figure 2** Association between identified genes and overall mortality in TCGA ccRCC (KIRC) dataset

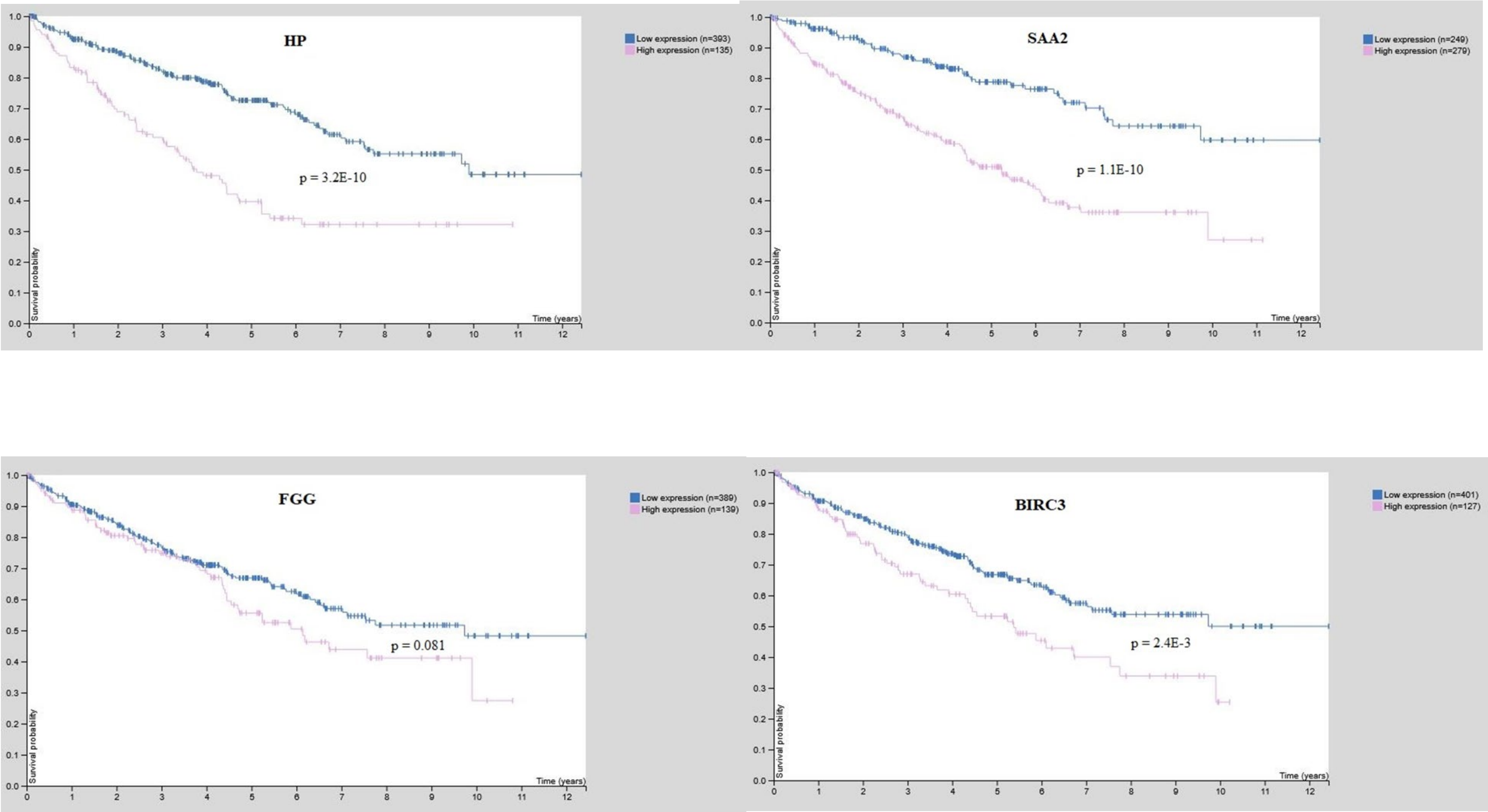

**Supplementary Figure 2** Association between identified genes and overall mortality in TCGA ccRCC (KIRC) dataset

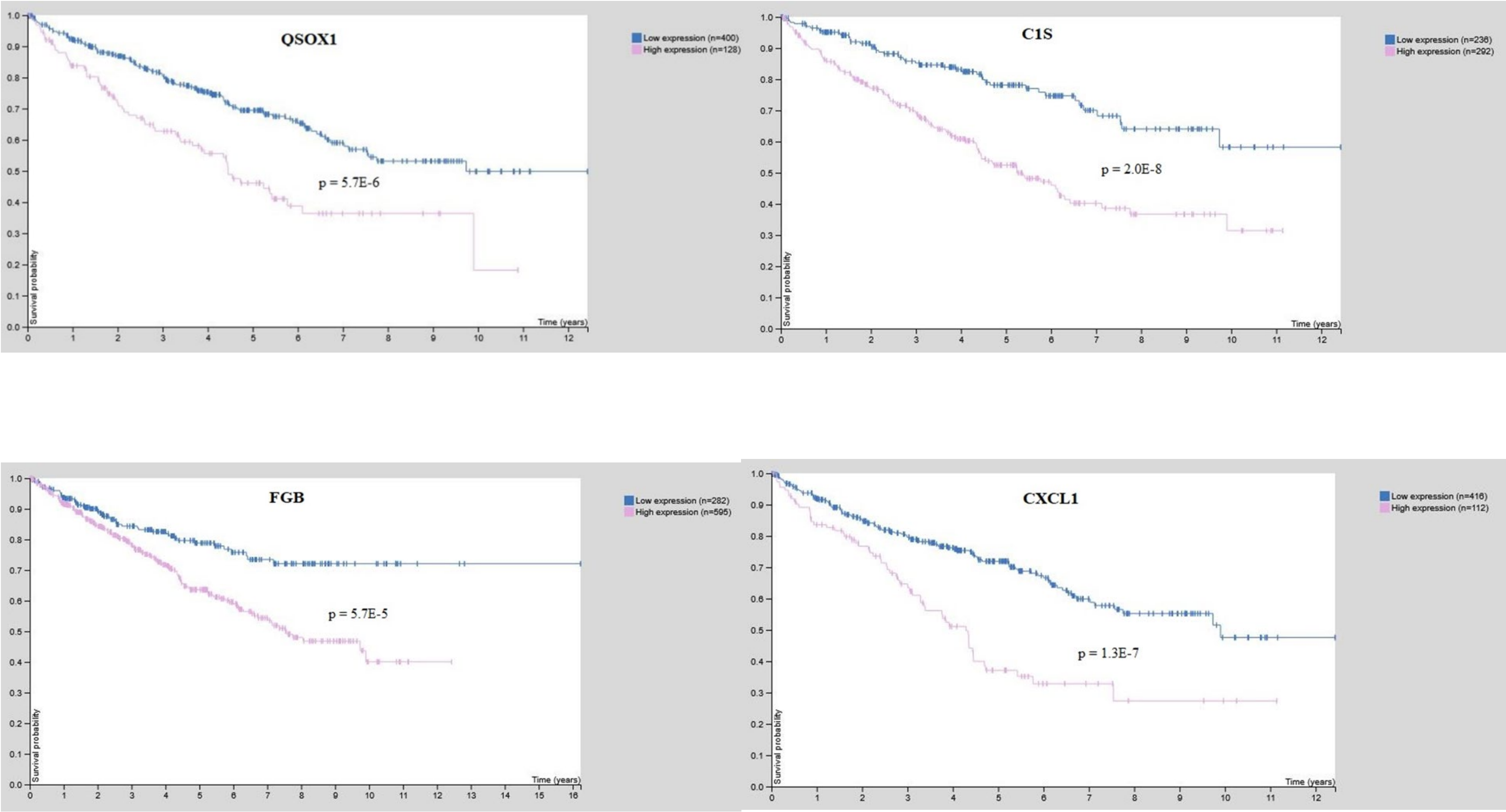

**Supplementary Figure 2** Association between identified genes and overall mortality in TCGA ccRCC (KIRC) dataset

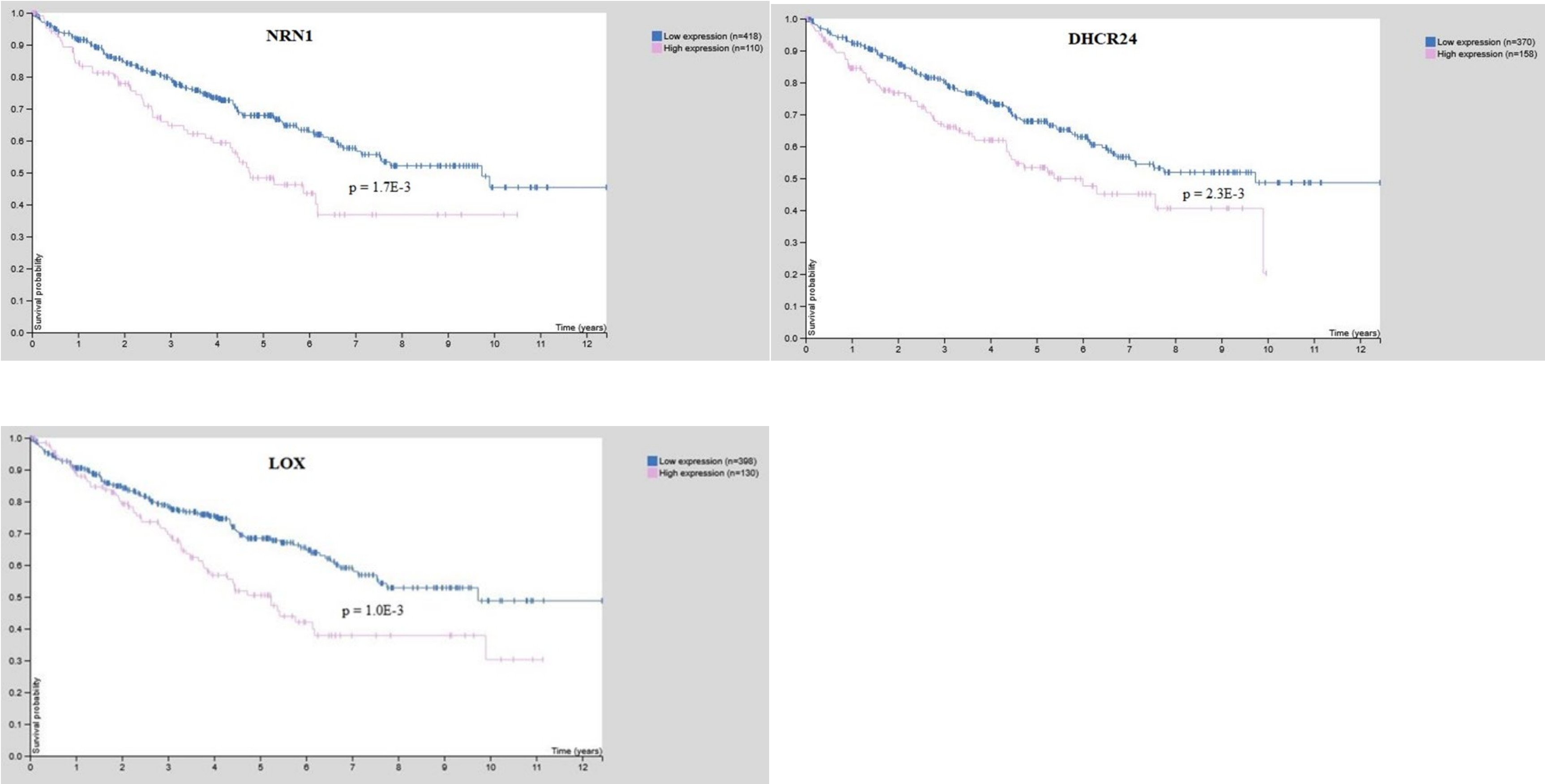

Supplement: Supplementary file 1 — Data S1: [file CAM4-12-12792-s001.pdf]
